# Plasma virome dynamics in chronic hepatitis B virus infected patients

**DOI:** 10.3389/fmicb.2023.1172574

**Published:** 2023-05-09

**Authors:** Marijn Thijssen, Frank Tacke, Lore Van Espen, David Cassiman, Mahmoud Naser Aldine, Frederik Nevens, Marc Van Ranst, Jelle Matthijnssens, Mahmoud Reza Pourkarim

**Affiliations:** ^1^Laboratory for Clinical and Epidemiological Virology, Department of Microbiology, Immunology and Transplantation, Rega Institute, KU Leuven, Leuven, Belgium; ^2^Department of Hepatology and Gastroenterology, Campus Virchow-Klinikum and Campus Charité Mitte, Charité - Universitätsmedizin Berlin, Berlin, Germany; ^3^Laboratory of Viral Metagenomics, Department of Microbiology, Immunology and Transplantation, Rega Institute, KU Leuven, Leuven, Belgium; ^4^Department of Gastroenterology and Hepatology, University Hospital Leuven, Leuven, Belgium; ^5^Health Policy Research Centre, Institute of Health, Shiraz University of Medical Sciences, Shiraz, Iran; ^6^Blood Transfusion Research Centre, High Institute for Research and Education in Transfusion Medicine, Tehran, Iran

**Keywords:** virome, hepatitis B virus, anellovirus, pegivirus, blood, plasma, metagenomic

## Abstract

The virome remains an understudied domain of the human microbiome. The role of commensal viruses on the outcome of infections with known pathogens is not well characterized. In this study we aimed to characterize the longitudinal plasma virome dynamics in chronic hepatitis B virus (HBV) infected patients. Eighty-five longitudinal plasma samples were collected from 12 chronic HBV infected individuals that were classified in the four stages of HBV infection. The virome was characterized with an optimized viral extraction protocol and deep-sequenced on a NextSeq 2500 platform. The plasma virome was primarily composed of members of the *Anello*- *Flavi*-, and *Hepadnaviridae* (HBV) families. The virome structure and dynamics did not correlate with the different stages of chronic HBV infection nor with the administration of antiviral therapy. We observed a higher intrapersonal similarity of viral contigs. Genomic analysis of viruses observed in multiple timepoint demonstrated the presence of a dynamic community. This study comprehensively assessed the blood virome structure in chronic HBV infected individuals and provided insights in the longitudinal development of this viral community.

## Introduction

Chronic infection with the hepatitis B virus (HBV) is one of the leading causes of liver cirrhosis and hepatocellular carcinoma (Schmit et al., 2021). Despite the availability of an effective vaccine and antiviral treatments, HBV remains a significant threat to public health. In 2016, a program was adopted by the World Health Assembly to eliminate viral hepatitis by 2030 (World Health Organization, 2016). Although countries have prioritized efforts to achieve these ambitious goals, the World Health Organization estimated that 257 million people are still living with chronic HBV infection globally (Pourkarim et al., 2018; Thijssen et al., 2019; World Health Organisation, 2020). HBV is considered a non-cytopathic virus that can cause long-lasting infections if the host immune system is not able to eliminate the virus (Iannacone and Guidotti, 2022). The induction of hepatic inflammation is caused by an immune response against infected hepatocytes. Individuals chronically infected with HBV may progress through different clinical stages that are characterized by a combination of well-established serum and liver markers (Tang et al., 2018). Most exposed individuals develop a self-limited infection and successfully eliminate the virus. However, individuals infected during infancy or early childhood have a higher tendency of developing chronic HBV infection (Yuen et al., 2018). Cycles of low-grade inflammation and reactivation of viral infection predisposes infected individuals for developing liver fibrosis and cirrhosis which might progress to decompensated liver disease and hepatocellular carcinoma (Yuen et al., 2018). Therefore, a better understanding of the viral-host interaction and effects of antiviral therapies in different stages of HBV infection could contribute to personalized treatment approaches and improved clinical control of disease.

Accumulating evidence supports a crucial role of the commensal microorganisms inhabiting the human body in health and disease, including HBV infection. For instance, it has been demonstrated that the maturation of microbiota in adult mice stimulates liver immunity, which supports a rapid clearance of HBV and resistance against other types of infectious or non-infectious injuries (Chou et al., 2015; Rosshart et al., 2017, 2019). Furthermore, fungal and bacterial members of the gut microbiota were related to disease progression in HBV patients (Chen et al., 2011; Wei et al., 2013; Liu et al., 2019). So far, research efforts have predominantly focused on the bacterial part of the microbiome, while the viral part (i.e., the virobiota) remains an understudied domain. The virobiota is considered the most abundant and genetically diverse fraction of the microbiome. By applying advanced sequencing technologies, studies of the virobiota collective genomes (i.e., the virome) revealed an important role of viral communities in the innate and adaptive immune responses (Focà et al., 2015). Interactions between the host and dwelling viruses can augment pathogen susceptibility as well as the response to vaccines by modulating the so-called immunophenotype (Virgin, 2014). As the pathogenic impact of HBV infection is caused by the complex interactions with the host immune system, understanding the virobiota and immune system interactions is of utmost importance. In this context, the role of ‘commensal’ viruses in chronic HBV infections is poorly understood. This study will focus on the characterization of the plasma virome in chronic HBV infected patients and highlights possible implications for these viruses in the clinical course of chronic HBV infection.

## Materials and methods

### Study population and ethics statement

Twelve patients were randomly selected from a cohort of HBV infected individuals that are followed in University Hospital (UZ) Leuven (Belgium). The patients were selected based on the clinical stage of chronic HBV infection and the availability of yearly longitudinal plasma samples that covered >4 years. Individuals coinfected with HIV or HCV were excluded. Based on clinical and para-clinical parameters, including molecular diagnostics and serology, these patients were classified in the different stages of chronic HBV infection. Chronic HBV infected patients can be classified in chronic HBV infection or chronic hepatitis B disease. Patients diagnosed with chronic HBV infection are subdivided in (I) HBeAg positive or (II) HBeAg negative, and chronic hepatitis B disease. Positive HBeAg indicates active replication of HBV, while the absence indicates minimal or no replication. Patients diagnosed with chronic hepatitis B disease are classified in (III) chronic hepatitis B or (IV) liver cirrhosis. In total, 85 samples were collected from the hospital biobank (stored at −20°C). An average of seven samples per patient were collected with a one-year interval. Biochemical, serological, and virologic markers were assayed by the hospital laboratories. Moreover, demographic data including age, sex, country of origin, and medication were obtained from the patient’s clinical record. This study was performed in accordance with the Declaration of Helsinki and was approved by the Ethics committee research UZ/KU Leuven, Belgium (S56121/ML10229). All participants gave informed consent and were able to withdraw from the study at any moment.

### Plasma virome sequencing

Viral particles enrichment was done according to the NetoVIR protocol adapted for plasma virome analysis (Conceição-Neto et al., 2015). Briefly, plasma samples were centrifuged at 17,000 g for 3 min and the supernatant was filtered through 5·0 μm polypropylene (Millipore) and 0·8 μm polyether sulphone filters (Sartorius). The filtrate was treated with a cocktail of micrococcal nuclease (New England Biolabs) and benzonase (Millipore) for 2 h at 37°C. Viral DNA and RNA were extracted with the QIAmp Viral RNA Mini kit (Qiagen) without carrier RNA. The Whole Transcriptome Amplification 2 kit (WTA2, Sigma Aldrich) was used for reverse transcription and 20 cycles of random amplification of the extracted nucleic acids. The amplified samples were purified with the MSB SPIN PCRAPACE kit (Stratec). Libraries were prepared for sequencing with the Nextera XT kit (Illumina) and quantified using the Qubit fluorometer (Thermo Fisher Scientific). The High Sensitivity DNA kit (Agilent) for the Bioanalyzer 2100 (Agilent) was used to determine the average library fragment size. Samples were pooled in equimolar ratio’s and paired-end sequencing was performed on the NextSeq 2500 platform (Illumina), with an average of 10 million reads (base length of 150) per sample.

### Read processing and taxonomical annotation

Reads were trimmed using Trimmomatic (v0.36) to remove WTA2 and Nextera XT primers as well as low quality (parts of) reads (Bolger et al., 2014). Both the 19 leading and tailing base pairs were removed. Subsequent trimming occurred using a sliding window of 4 base pairs with an average cut-off PHRED score of 20 and a minimal read size of 50 base pairs. Following the removal of human genome and contaminating sequences with Bowtie2 (v2.3.4.1), metaSPAdes (v3.11.1) was used for *de novo* assembly of contigs using kmer sizes of 21, 33, 55, and 77 bp (Langmead and Salzberg, 2012; Nurk et al., 2017). Contigs from all samples with a length above 500 bp were clustered at 80% coverage and 95% nucleotide identity using nucmer from the MUMmer package (v3.23) to construct a non-redundant dataset (Bolduc, 2017; Simon and Ben, 2017). Individual reads were mapped to the non-redundant contig database using bwa2 (v2.0) and a cut-off of 70% coverage was used for the presence of contigs (Vasimuddin et al., 2019). The classification of contigs was done using ktClassifyBLAST on DIAMOND protein hits (database downloaded March 2021) and with BLASTn (E-value of 1e-10) against the NCBI nt database (downloaded March 2021) (Altschul et al., 1990; Ondov et al., 2011; Buchfink et al., 2015). For bacteriophage identification, VirSorter2 (v1.1.0) was used and CheckV (v0.8.1) to assess the completeness of viral genomes (Guo et al., 2021; Nayfach et al., 2021).

### Phylogenetic analysis

For the anelloviruses, open reading frame 1 (ORF1) protein sequences were predicted with the “getorf” function in the EMBOSS software package (Rice et al., 2000). The translated non-redundant ORF1 protein sequences were aligned with anellovirus reference sequences (271 sequences downloaded from NCBI database, November 2021) using MAFFT (v7.407) with the iterative refinement method employing both the WSP and consistency scores (Katoh and Standley, 2013). The aligned sequences were trimmed using trimAI (v1.1, gappyout setting) (Capella-Gutiérrez et al., 2009). RAxML (v8.2) was used under model VT + I + G4 + F determined by jModelTest (v2.1.10) with 1,000 bootstrap replicates to build the phylogenetic tree (Darriba et al., 2012; Stamatakis, 2016). The pegivirus phylogenetic tree was built with the non-structural protein 5B (NS5B, RNA-dependent RNA polymerase) sequences extracted from the dataset aligned with available NS5B pegivirus reference sequences (46 sequences downloaded from NCBI RefSeq, November 2021). The tree was built under model LG + G4 + F determined by jModelTest (v2·1·10) in RAxML (v8.2) with a 1000 bootstrap replicated (Darriba et al., 2012; Stamatakis, 2016). The HBV tree was constructed based on the whole genome sequences of our dataset and reference sequences (50 sequences downloaded from NCBI database, March 2021) of different genotypes. MAFFT (v7·407) was used to align the sequences with the iterative refinement method employing both the WSP and consistency scores (Katoh and Standley, 2013). A neigbor-joining tree was built from the aligned dataset with 1,000 bootstrap replicates in MEGA (v7.0) (Kumar et al., 2016). The trees were visualized and annotated with the ggtree package (v3.0.4) (Yu, 2020).

### Statistical analysis

All analyses were performed using R-software (v4.1.1) (Team, 2013). The annotated datasets, excluding HBV viral reads, were subsampled to a depth of 198,646 reads using the “rarefy_even_depth” function of the phyloseq package (v1.34.0) (McMurdie and Holmes, 2013). Mann-Whitney U test was applied to explore differences between two continuous variables. For testing of multiple groups, the Kruskal-Wallis test was deployed and where significant differences were observed, a *post-hoc* Dunn’s test was used with Bonferroni correction for multiple testing. Correlations between variables were expressed by the Spearman’s rank correlation coefficient. The vegan package (v2.5-7) was used for computing the alpha- and beta-diversity, and for performing a distance-based redundancy analysis (dbRDA) with the “dbrda” function (Oksanen et al., 2007). Function “ordiR2step” was applied for stepwise model building in the dbRDA analysis. Importing the aligned fasta sequences and performing the consecutive PCA analysis was done with the bios2mds package (v1.2.3) (Pelé et al., 2012). Finally, figures were plotted with the ggplot2 package (v3.3.5) (Wickham, 2016).

## Results

### Patient characteristics

**Figure 1:**
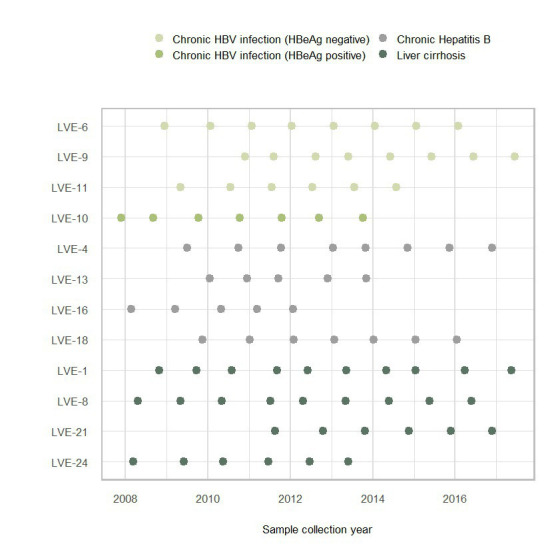
Sample collection figure categorized according to the four stages of chronic HBV infection.

The overall patient characteristics and clinical data are shown in Table 1. Twelve chronic HBV infected patients were included in the study with a median age of 48 years at the time of inclusion. These patients were categorized according to the current EASL guidelines for nomenclature of chronic hepatitis B stages (Liver, 2017). Based on this classification, patients were grouped into chronic HBV infection (*N* = 4, one HBeAg positive and three HbeAg negative patients) and chronic hepatitis B disease (*N* = 8, four chronic hepatitis B and four liver cirrhosis patients). All patients with chronic hepatitis B disease received antiviral treatment, with single or combined drugs. Antivirals were prescribed based on an assessment of patients’ background data and clinical parameters (e.g., HBV DNA levels and severity of liver disease determined by hepatological markers). Plasma samples were collected with an average interval of 1 year, depending on the availability of samples in the hospital blood bank (Figure 1). In total, 85 samples were obtained (average of seven samples per patient) and sequenced to characterize the plasma virome community.

**Table 1:** Demographic characteristics of the study population.

Patient ID	Age	Gender	Stage	Geographical region
LVE-1	48	Male	Liver cirrhosis	Middle East
LVE-4	49	Male	Chronic hepatitis B	Europe
LVE-6	57	Male	Chronic HBV infection (HBeAg negative)	Europe
LVE-8	36	Male	Liver cirrhosis	Africa
LVE-9	31	Male	Chronic HBV infection (HBeAg negative)	Europe
LVE-10	30	Male	Chronic HBV infection (HBeAg positive)	Asia
LVE-11	48	Female	Chronic HBV infection (HBeAg negative)	Europe
LVE-13	32	Male	Chronic hepatitis B	Africa
LVE-16	60	Male	Chronic hepatitis B	Europe
LVE-18	53	Female	Chronic hepatitis B	Asia
LVE-21	62	Male	Liver cirrhosis	Europe
LVE-24	37	Male	Liver cirrhosis	Europe

### Virome characterization

Illumina sequencing resulted in a total number of ~684 million reads. Samples were sequenced on an average depth of ~8 million reads (sd: + 1.5 million reads). In total, ~220 million reads were used for *de novo* assembly after quality trimming and contamination removal (experimental and host contamination). In total, 26 million reads were annotated as viral, both eukaryotic and prokaryotic viral reads, which corresponded to 3.4% (mean 3.6 + 0·07%) of the total number of generated reads. The majority of eukaryotic viral reads were attributed to three viral families, including the *Anello*-, *Flavi*-, and *Hepadnaviridae* (Supplementary Figures S1A,B). In terms of absolute number of reads, the *Hepadnaviridae* were most abundant followed by the *Flaviviridae* and *Anelloviridae*. However, the overall number of patients positive for specific viral families was highest for the *Anelloviridae* (*N* = 11) followed by *Hepadnaviridae* (*N* = 9) and *Flaviviridae* (*N* = 3). Besides these three abundant families, other viral families (e.g., low number of *Totiviridae* reads) represented only a small fraction (<0.5%) the total amount of reads.

### The plasma virome in different clinical stages of chronic HBV infection

**Figure 2:**
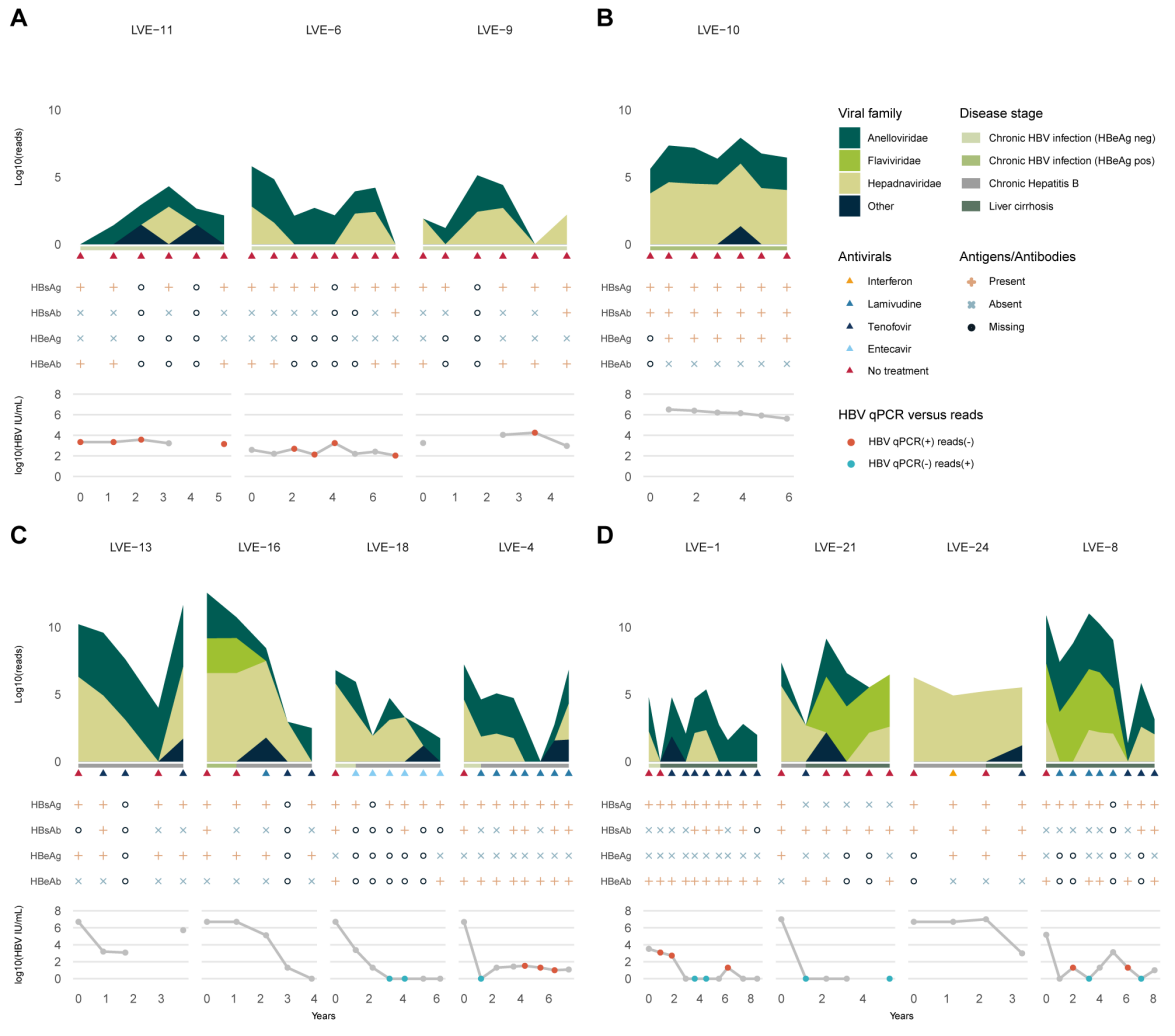
Evolution of the eukaryotic viral families, HBV antibodies and antigens, and HBV viral load. **(A)** Chronic HBV infected (HBeAg negative), **(B)** chronic HBV infected (HBeAg positive), **(C)** chronic hepatitis B and **(D)** liver cirrhosis patients. Colored dots indicate discrepancies between the qPCR and data and presence of HBV reads. Red dots: (+) qPCR and (−) HBV reads. Blue dots: (−) qPCR and (+) HBV reads. HBsAg, hepatitis B a antigen; HBsAb, hepatitis B s antibody; HBeAg, hepatitis B e antigen; HBeAb, hepatitis B e antibody.

The patients were classified in chronic HBV infection (HBeAg positive or negative), and chronic HBV disease (chronic hepatitis B or liver cirrhosis) according to the EASL guidelines. Three patients were diagnosed with HBeAg negative chronic HBV infection at the moment of inclusion (Figure 2A). Based on the clinical and serological parameters, all three patients did not progress to chronic HBV disease, and therefore, did not require antiviral therapy. Although the laboratory PCR data demonstrated a relatively stable HBV viral load (LVE-6 2.0–3.2, LVE-9 3.0–4.3, and LVE-11 3.2–3.6 log HBV IU/mL), the sequencing data showed variations in the virome composition. In contrast to HBV, reads belonging to members of the *Anelloviridae* remained relatively stable, although they were absent in some samples.

One patient (LVE-10) was diagnosed with HBeAg positive chronic HBV infection and was not treated with antiviral medication (Figure 2B). In line with the stage classification of this patient, the abundance of HBV reads was consistently high during follow-up. This observation corroborates the laboratory findings that demonstrated a high HBV viral load in this patient (range 5.6–6.5 log HBV IU/mL). Besides the HBV, a small fraction of the virome consisted of the *Anelloviridae*.

Four patients were classified with chronic hepatitis B disease (Figure 2C). Except for patient LVE-13, all patients progressed from the chronic HBV infection stage (HBeAg positive or negative) to chronic Hepatitis B during follow-up. The progression to this stage required the initiation of antiviral therapy, including lamivudine, entecavir, and tenofovir. In three patients (LVE-16, 18, and 4) an overall decreasing trend is observed in the number of HBV reads and qPCR data. However, in patient LVE-16 the HBV viral load and sequencing data did not substantially decrease from timepoint 2 to 3, even though antiviral therapy was administered. This patient reported lamivudine resistance and switched the antiviral therapy to tenofovir. This therapy adjustment caused a drop in the HBV read abundance as well as HBV viral load. In patient LVE-13, pregnancy urged a therapy break in timepoint 4 resulting in a flare of HBV reads. Besides the presence of HBV, the virome largely consisted of members of the *Anelloviridae* and a member of the *Flaviviridae* (human pegivirus) in patient LVE-16.

The final group included four patients that were diagnosed with liver cirrhosis (Figure 2D). Both patients LVE-21 and 24 progressed from the chronic hepatitis B stage to liver cirrhosis, while patient LVE-1 progressed from the HBeAg negative stage. All cirrhosis patients received a variety of antiviral medication to actively suppress HBV, including interferon, lamivudine, and tenofovir. Patient LVE-24 had a stable presence of HBV both in terms of reads and qPCR viral load. Other patients demonstrate variable abundances for HBV. Interestingly, discrepancies were observed between the sequencing and qPCR results, with samples positive for HBV reads but negative in qPCR in patients LVE-1, LVE-8, and LVE-21. Besides the widely abundant HBV and anelloviruses, two cirrhosis patients reported the presence of human pegiviruses (*Flaviviridae*). The clinical data indicated that both patients experienced regression of liver cirrhosis during follow-up. This regression was reported based on a decreased METAVIR score determined by transient elastography (fibroscan).

### Characterization of the widely abundant members of the *Anelloviridae* family

**Figure 3:**
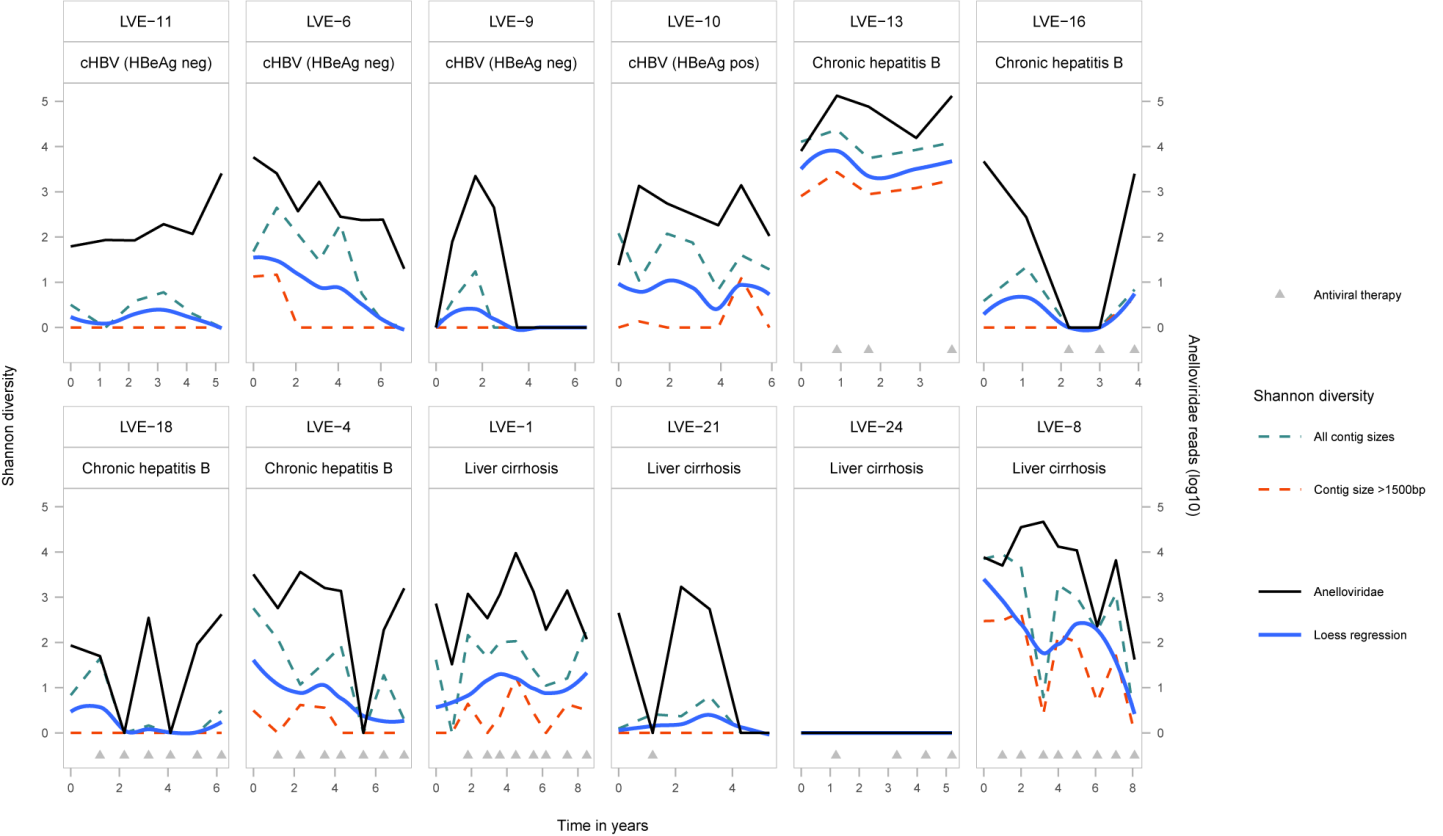
Alpha-diversity (Shannon-diversity index) evolution of the *Anelloviridae* community per patient. The Shannon diversity index is displayed at the left axis and the anellovirus abundance on the right axis. Shannon diversity is calculated based on two different dataset (1) including all anellovirus contigs (blue dotted line) and (2) including contigs with a size above 1,500 bp (red dotted line). A loess regression line (blue line) illustrates the Shannon diversity trend based on the two datasets.

Viruses belonging to the *Anelloviridae* family were most widely detected in the studied samples. A total of 66 (77%) samples from 11 (92%) patients were found positive for anelloviruses. To determine population differences and the evolution of the anellovirus population, we calculated the alpha diversity dynamics from the non-redundant OTU database including all contigs annotated as anelloviruses, as well as a subset containing only contigs with a size above 1,500 bp (Figure 3). A regression line was added to visualize the Shannon diversity trend between the two datasets. The inclusion of contigs with a size above 1,500 bp, which avoids the counting of multiple parts of a single genome as different species, causes a substantial drop in alpha diversity. Even though some samples have a substantial number of anellovirus reads, the assembled contigs only represent shorter fragments (<1,500 bp). Only 13.9% of the observed contigs have a size above 1,000 bp (Supplementary Figure S2). The community diversity did not correlate with specific stages of HBV infection nor the administration of antiviral therapy (Kruskal-Wallis test: *p* > 0.05). Remarkably, no anellovirus reads were observed in patient LVE-24. The alpha diversity correlated with the abundance of *Anelloviridae* reads in both datasets (Spearman’s rho = 0.76 and 0.74, *p* < 0.001, for the complete and 1,500 bp datasets respectively).

**Figure 4:**
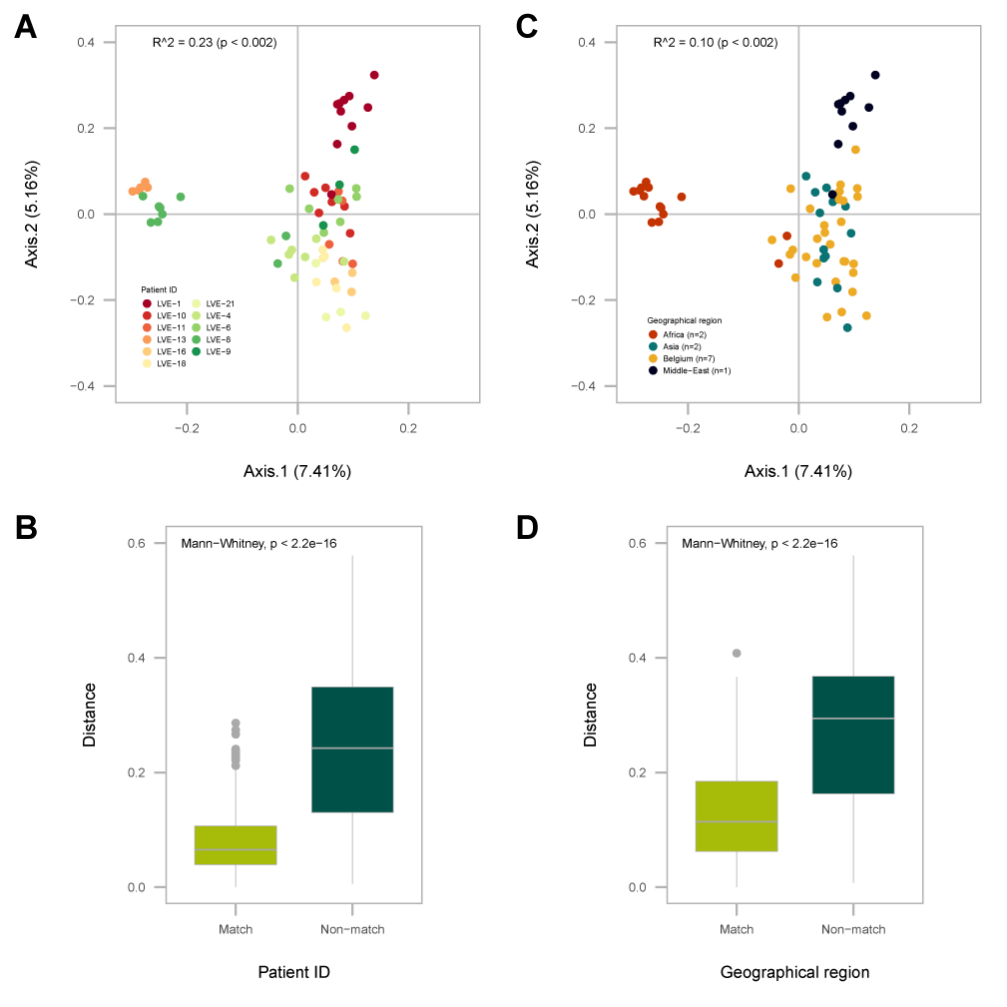
Analysis of the Bray-Curtis dissimilarity (beta-diversity) calculated on the presence and absence of *Anelloviridae* OTUs. **(A)** PCA plot of the distance based redundance analysis (dbRDA) with sample points colored according to patient ID. **(B)** Overview of the intra- (match) and inter-patient (non-match) distances between individual sample points. **(C)** PCA plot of the dbRDA with sample points colored according to the patient geographical origin. **(D)** Distances between individual sample points of patients originating from the same geographical region (match) or other regions (non-match). Significance was determined with a univariate dbRDA analysis. Distances were compared with the Mann-Whitney U test.

A dbRDA analysis was performed on a Bray–Curtis dissimilarity matrix of the presence and absence of anellovirus OTUs (Figure 4). Two explanatory variables significantly contributed to the clustering of samples in the biplot. Firstly, we observed the clustering of samples based on patient ID (*R*^2^ = 0.23, univariate dbRDA, *p* < 0.002), meaning that samples from the same patient had a more conserved anellovirus virome composition compared to samples from different individuals (Figure 4A, *R*^2^ = 0.23, *p* < 0.005 and Figure 4B, Mann–Whitney U test, *p* < 0.05). In addition, geographical origin of the patient also contributed to the clustering of samples (Figure 4C, *R*^2^ = 0.10, univariate dbRDA *p* < 0.002, and Figure 4D, Mann–Whitney U test, *p* < 0.05). Remarkably, two patients originating from Africa, both of Cameroonian origin, formed a separate cluster (Figure 4C), suggesting that these patients have more similar anellovirus communities compared to other individuals. Furthermore, a patient from the Middle Eastern region clustered separately from Belgium and Asian patients, with the latter two groups demonstrating a more dispersed ordination. However, a multivariate analysis modelling both patient ID and geographical origin showed the loss of statistical significance of geographical origin, indicating that the observed effect of geographical region is confounded by patient ID (stepwise dbRDA, *p* > 0.05).

**Figure 5:**
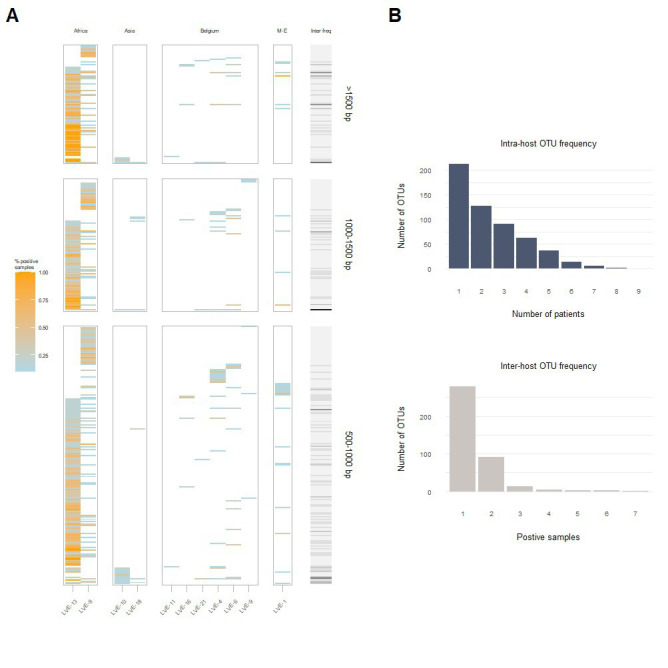
Intra- and inter-host composition of the anellovirus community. **(A)** A heatmap that demonstrates the presence and absence of contigs within patients and the percentage of samples positive for the respective contig (colored coded). The right bar (grey to black) corresponds to the number of patients positive for this contig. **(B)** The blue histogram indicates the number of contigs that were observed once or more within a single patient (top). The grey histogram demonstrates the number of contigs that appeared only in one patient or were shared between two or more patients (bottom). M-E, Middle East; OTU, operational taxonomical unit.

A heatmap reflecting the presence of individual contigs assigned to the *Anelloviridae* in different patient samples, demonstrates that most contigs are found among the two African patients (LVE-13 and LVE-8; Figure 5A). Analysis of the OTU frequency within and between patients indicate that the anellovirus community is more stable within compared to between patients (Figure 5B). The majority of OTUs are only present in a single patient, while some are present in two and up to seven patients. In contrast, OTUs are more often shared between samples of the same patient, indicating a more preserved intra-host anellovirus community.

**Figure 6:**
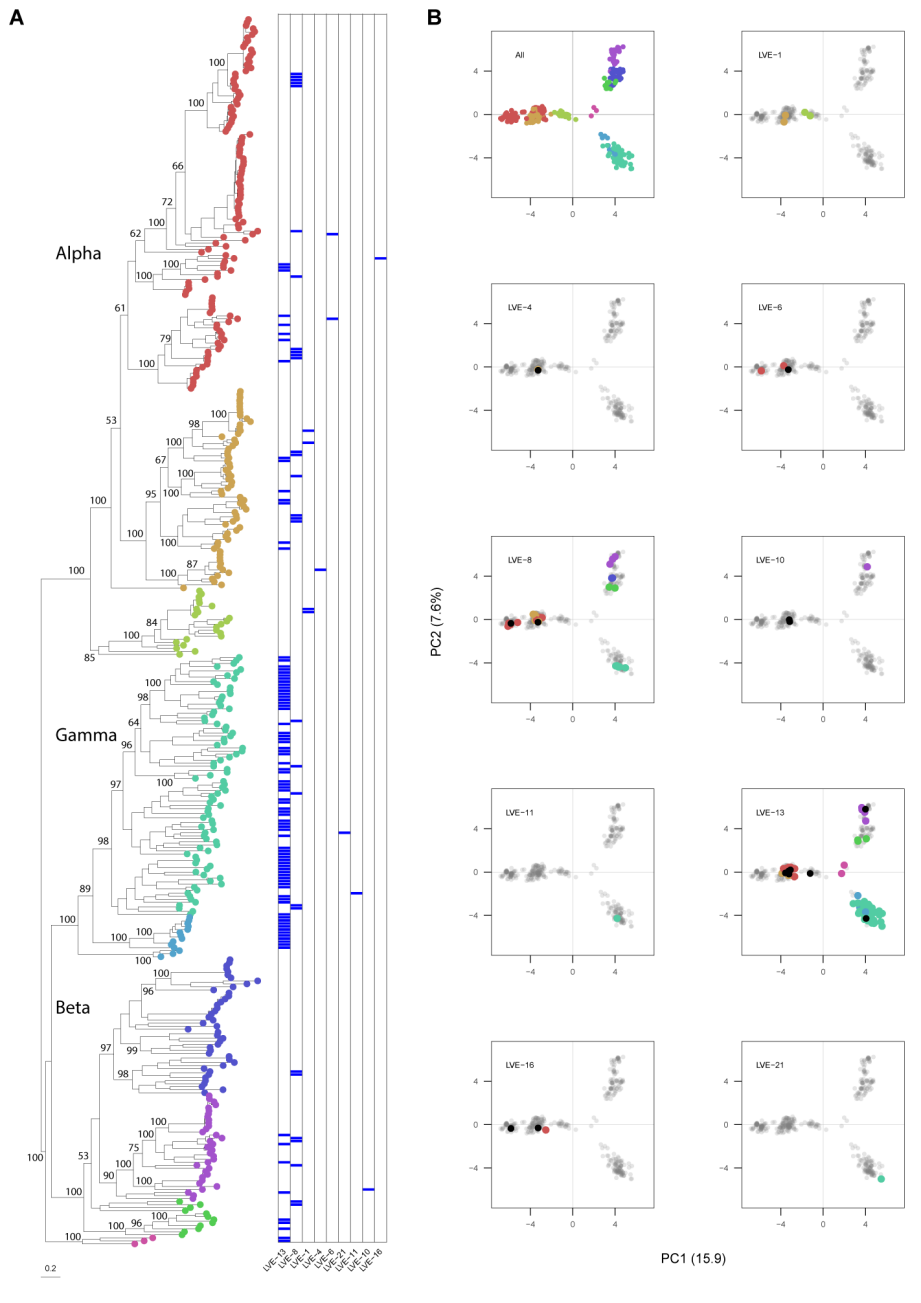
Phylogenetic analysis of the anellovirus ORF1 sequences and PCA analysis of the aligned dataset. **(A)** Maximum-likelihood tree of the patient and reference protein ORF1 sequences built in RAxML under model VT + I + G4 + F determined by jModelTest, with 1,000 bootstrap replicates. The heatmap illustrates positive viruses for the respective patient. Bootstrap values above 50 are shown for larger clades. **(B)** Principal components analysis of the aligned sequenced used for building the phylogenetic tree. Contigs extracted from patient samples are colored according to the tree position. Black dots indicate the presence of this contig based on 70% coverage, meaning that this contig was not derived from this patient but classified as present based on 70% coverage cut-off.

A maximum likelihood phylogenetic tree was constructed from the *Anelloviridae* ORF1 protein sequences extracted from the assembled contigs (*N* = 135) and reference databases (Figure 6A). The complete ORF1 sequences extracted from the patient samples clustered according to the clades of the three established genera within the *Anelloviridae*, which are known to infect humans (alpha-, beta-, and gamma-torquevirus). A PCA analysis was applied on the aligned ORF1 sequences to explore the evolutionary space occupied by the observed anellovirus sequences per patient (Figure 6B). In most patients the sequences only cover a single cluster in the PCA plot, i.e., corresponding with a single anellovirus genus. In contrast, in patients LVE-8 and LVE-13 (both of African origin) sequences were observed that occupy a large evolutionary space. Besides the ORF1 sequences directly extracted from corresponding patients, mapping of the reads to the clustered patient ORF1 sequences demonstrated the presence of additional sequences (70% horizontal coverage cut-off). These additional sequences were primarily derived from the similar clusters except for patients LVE-4 and LVE-13.

**Figure 7:**
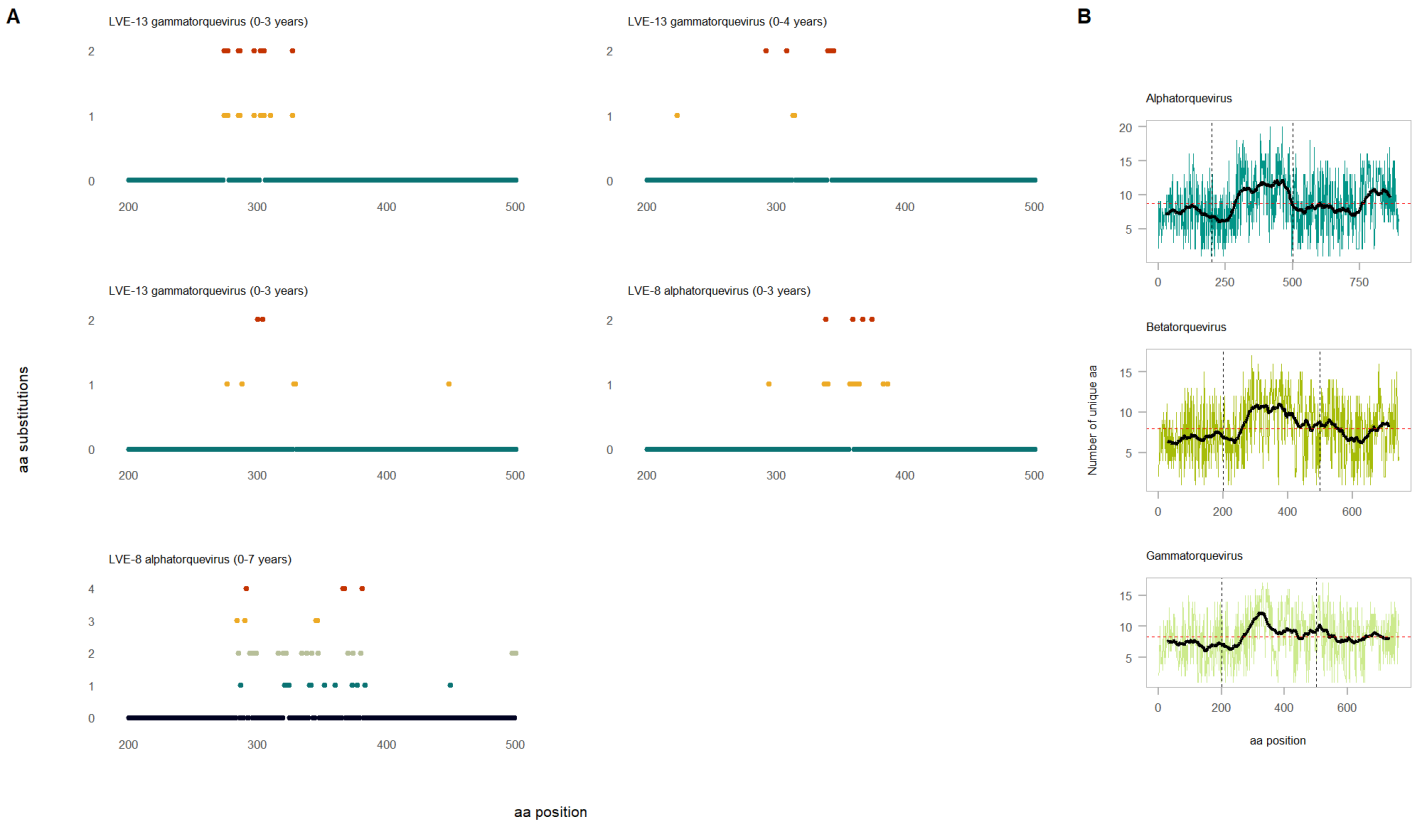
Evolution of the anellovirus contigs observed at multiple timepoints. **(A)** Amino acid diversity of ORF1 sequences that were observed in multiple timepoint (amino acid position 200–600). Colored dots indicate amino acid substitutions compared to the reference sequence observed in the earliest timepoint. The color code corresponds to the substitution frequency over time. **(B)** Diversity of the ORF1 region based on the extracted sequences and reference sequences for the respective genus. The horizontal red dotted line indicates the average number of unique amino acid over the entire ORF1. The vertical lines highlight the hyper variable region.

To study the intra-host genetic evolution of the anellovirus community, contigs with a high amino acid similarity that were present in multiple timepoint were extracted and aligned (>90% amino acid similarity, Supplementary Figure S3 and Figure 7A). Highly identical sequences observed in multiple timepoints were retrieved from both patient LVE-8 and LVE-13 (Figure 7A). The alignment demonstrates that amino acid substitutions occur frequently over time. Most substitutions occurred between amino acid position 200–500, the hyper variable region (HVR) of the anellovirus genome, independent of the anellovirus genus. The alignment of all ORF1 sequences, including reference sequences, confirm the presence of the HVR between amino acid position 200–500, albeit variations can be found across the entire ORF1 gene (Figure 7B).

### Analysis of the *Hepadnaviridae* (HBV) sequences

**Figure 8:**
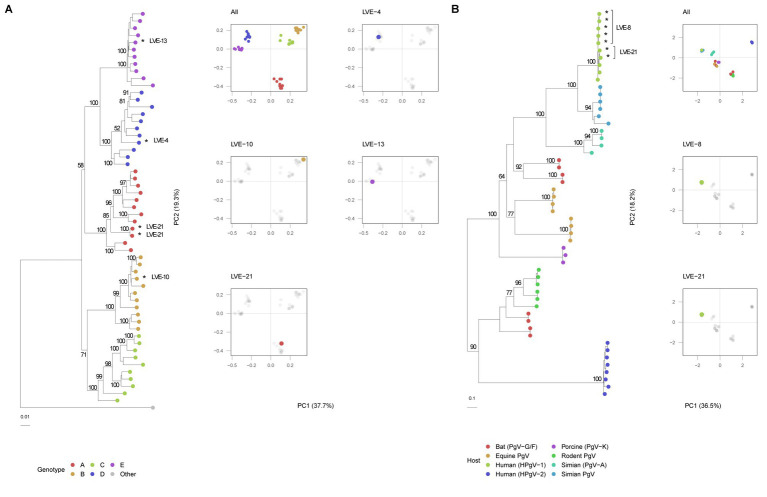
Phylogenetic and PCA analysis of the identified NS5B pegivirus regions and HBV genomes. **(A)** HBV neighbor-joining tree with 1,000 bootstrap replicates and PCA analysis of the aligned sequences. A Woolly monkey HBV genome was used as an outgroup (AY226578.1). Bootstrap values above 50 are shown for larger clades. **(B)** Maximum-likelihood tree of the pegivirus NS5B sequences extracted from patient samples and reference database of different hosts. The tree was built under model LG + G4 + F determined by jModelTest with 1,000 bootstrap replicates. Bootstrap values above 50 are shown for larger clades. *Indicates patient derived sequences.

The *Hepadnaviridae* family consumed most of the sequenced viral reads and were widely present across different individuals (*N* = 9). All reads attributed to this viral family were annotated as HBV. Of the 57 samples that were positive for HBV qPCR, 40 samples were confirmed with the applied sequencing method (corresponding to a sensitivity of 70%). Remarkably, nine samples positive for the sequencing method were negative for qPCR. Overall, a significant correlation was observed between the HBV sequencing reads and qPCR (Spearman’s rho = 0.60, *p* < 0.001). In total, four complete HBV genomes were assembled from samples of different patients (Table 2). A neighbor-joining tree was constructed using the assembled HBV genomes and representative HBV sequences of the known HBV genotypes with 1,000 bootstrap replicates (Figure 8A). The HBV genomes in our study population belonged to genotype A, B, D, and E. The PCA analysis illustrates the clustering of similar genotypes (Figure 8A). In contrast to anelloviruses, only single genotypes were observed in the patient samples. These genotypes corresponded with the geographical dispersal of HBV and patients’ geographical origin (Table 2).

**Table 2:** Observed HBV genotypes in the study population and the patient country of origin.

Patient	HBV genotype	Country of origin
LVE-4	D	Belgium
LVE-10	B	China
LVE-13	E	Cameroon
LVE-21	A	Belgium

### Presence of *Flaviviridae* in chronic HBV infected patients

**Figure 9:**
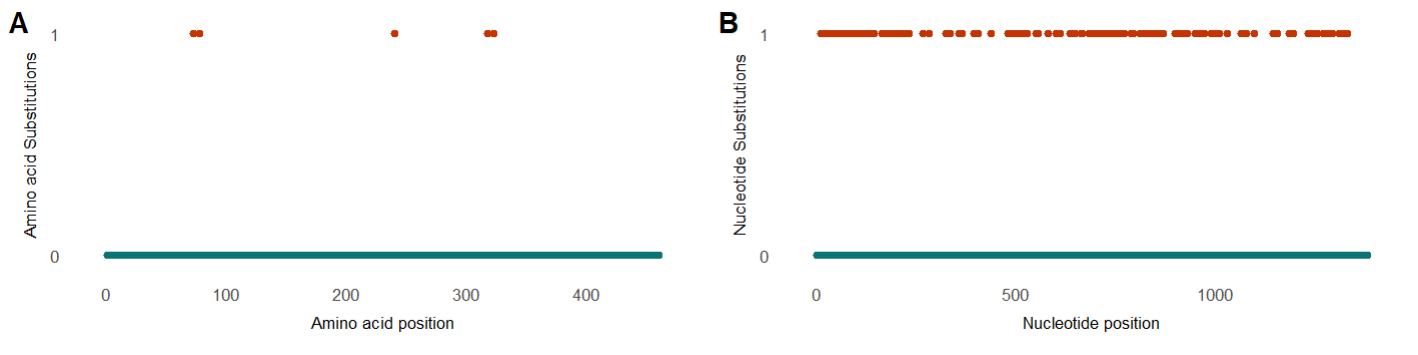
Evolution of the pegivirus NS5B region between patients **(A)** Alignment of the protein NS5B and **(B)** nucleotide sequences observed in two patients. The color code corresponds to the amino acid or nucleotide substitutions between NS5B sequences of both patients.

Three patients were found positive for viruses of the *Flaviviridae* family. The presence of this family was fully attributed to human-pegivirus-1 (HPgV-1). HPgV-1 sequences were observed in patients classified in the chronic hepatitis B and liver cirrhosis stages. Intact protein sequences of NS5B (*N* = 7, patients LVE-8 and LVE-21), the RNA-dependent-RNA polymerase, were extracted from the sequencing datasets and used to build a maximum likelihood phylogenetic tree (Figure 8B). The NS5B sequence observed in the study population formed a clade with reference sequences of HPgV-1, Pegivirus-C (formerly known as GB virus C). We performed a PCA analysis on the aligned NS5B amino acid sequences including reference sequences (Figure 8B). The NS5B regions extracted from the patient samples share a similar cluster in the PCA plot. In contrast to the anellovirus ORF1, this region of the pegivirus seems more stable across multiple timepoint (Supplementary Figures S4A,B). This region remained conserved on both amino acid (Supplementary Figure S4A) and nucleotide level (Supplementary Figure S4B) in two patients. Apart from a single nucleotide substitution in NS5B sequences of patient LVE-21, no further changes were observed in both amino acid and nucleotide sequences within a single individual. However, the comparison of sequences of both patients, showed multiple amino acid and nucleotide substitutions (Figures 9A,B). The nucleotide substitutions between patient sequences were mostly synonymous that did not alter the amino acid sequence, with the exceptions of five amino acids.

## Discussion

In this study, we have evaluated the plasma virome dynamics in chronic HBV infected patients and sought to elucidate the clinical relevance of these viral communities. We have applied an optimized method for intact viral particle extraction and used a shotgun metagenomic approach to sequence the viruses circulating in the blood of patients. The virome constituted viruses of well-known bloodborne viral families and displayed a considerable diversity among different patients. No clear associations were observed between the presence of specific viruses and stages of HBV disease. A detailed analysis of the anellovirus community revealed a personalized population structure with little sharing of genome sequences. Furthermore, a remarkable diversity of anellovirus contigs were observed in two African patients, which might imply a potential effect of geographical origin.

The metagenomic analysis allowed us to investigate the viral landscape in HBV infected individuals. The application of NGS methods in the clinic for routine diagnosis of viral infections is still under debate. Although, NGS has the ability to identify novel and rare infections, it is still labor-intensive and has lower sensitivity compared to targeted techniques such as qPCR, which could be an issue for samples with a low viral load (Perlejewski et al., 2020). Despite this, we observed a correlation between the laboratory qPCR results and metagenomic sequencing data, which demonstrated the robustness of our method. The metagenomic approach used in this study is developed to isolate nucleic acids from intact viral particles, while the qPCR method will also detect non-encapsidated viral genomes. This might underlie the observed discrepancies between the results of both methods. Furthermore, the untargeted nature of the viral metagenomic method is more susceptible to environmental and host contamination that will interfere with the detection of viruses. For the clinical application of NGS, standardized protocols for experimental and data analysis are highly recommended.

In terms of diversity, the viruses of the *Anelloviridae* family dominated the eukaryotic virome in chronic HBV infected individuals. Based on previous reports, anelloviruses are widely spread among the healthy population (Kaczorowska and van der Hoek, 2020). In total, we observed a positivity rate of 77% in all samples. Previous studies that used qPCR methods to detect anelloviruses reported a positivity rate ranging from 58 to 76% (Redondo et al., 2022). We expect that this percentage is an underestimation of the true prevalence, since the high diversity of anellovirus sequences hampers the development of universal anellovirus primers. Some studies have applied-pre amplification steps, including rolling circle amplification with or without anellovirus specific primers, to improve the sensitivity towards anelloviruses (Arze et al., 2021). However, these methods might negatively affect the detection of other viruses and could introduce substantial amplification bias. Furthermore, despite pre-amplification, even these studies report negative anellovirus samples in healthy individuals. A concern with untargeted metagenomic sequencing could be associated with high viral load of specific viruses (e.g., HBV). For instance, abundant viruses can consume the majority of generated reads, which decreases the likelihood of detecting low viral load viruses. However, even in samples with a high HBV load and number of reads, we were able to retrieve information on other viruses including anello- and pegivirus (e.g., in patient LVE-16). Therefore, we do not expect that these limitations underlie the absence of anelloviruses in some cases. Another possible explanation for the absence of anellovirus could be associated with overall quality of the biological samples.

Although, the presence of anellovirus has been associated to a variety of clinical conditions in the past, causal links to human disease have been lacking so far (Spandole et al., 2015; Reshetnyak et al., 2020). Previously, we have demonstrated the high prevalence of anelloviruses in liver transplant recipients, which corresponded with the initiation of immunosuppressive therapy (Thijssen et al., 2020). These findings suggested that the *Anelloviridae* abundance could be used as a surrogate marker of immunocompetence. Furthermore, previous studies hypothesized the use of anellovirus abundance as a marker for inflammation (Freer et al., 2018; Giacconi et al., 2018; Kaelin et al., 2022). Here, we were unable to link anellovirus dynamics to chronic HBV clinical course and disease severity. Even though, two patients (LVE-8 and LVE-13) showed a high anellovirus diversity, there were no major differences in absolute abundance between individuals. The lack of evident virome-disease associations could be related to the underlying patient diversity in our study population and the low sample size. Our results call for a better understanding of the impact of external variables on the virome structure and the potential clinical consequences for HBV infection.

Most anellovirus contigs were only observed in single patients and were not shared in the population. This suggests the presence of personalized virome structures, which confirms previous findings in virome studies that covered other niches of the human body (Shkoporov et al., 2019; Van Espen et al., 2021). Despite host individuality, additional variables contribute to variations in microbiome composition (Zuo et al., 2020). For instance, geographical origin of the patient seemed to contribute to the blood virome composition, albeit the observed effect was confounded by patient ID and restricted to a small study population. A previous study did observe minor differences in viruses found in women originating from different regions in China (Liu et al., 2018). The effect of environment on the microbiome composition has been more pronounced in the gut environment. For instance, perturbations of the gut microbiome have been reported in immigrants shortly after arriving in the United States, indicating a more versatile community (Vangay et al., 2018). Since the present study only describes virome dynamics in a limited population of chronic HBV infected individuals, it remains to be investigated whether similar variables affect the blood virome composition.

A recent paper demonstrated that the accumulation of non-pathogenic viruses, including anellovirus and pegivirus, preceded the acquisition of pathogenic viruses like HCV in people who inject drugs (Kandathil et al., 2021). This suggests that the transmission and accumulation of non-pathogenic viruses might be associated with the engagement in risk behavior (e.g., injecting drugs or unprotected sexual intercourse) (Yu et al., 2022). Two patients with both a history of syphilis infection, demonstrated a high diversity of anellovirus contigs and coinfection with pegivirus in one individual. These findings might suggest that the blood virome in these patients reflects an increased exposure to non-pathogenic viruses and the possible risk of acquiring infections with other pathogens. However, our study population is too small and we lack sufficient patient background data to support significant conclusions.

We were able to retrieve intact anellovirus ORF1 sequences from all three established genera within the *Anelloviridae*, which are known to infect humans. Based on the phylogenetic analysis, most patients had contigs from a single genus, while three patients reported sequences derived from multiple genera. Furthermore, the anellovirus contigs observed in patients from African origin covered the highest evolutionary space that included all three genera. These observations confirm previous data that demonstrated the co-infections with multiple genera in single individuals (Al-Qahtani et al., 2016). Diversity of the anellovirus ORF1 is predominantly concentrated in the HVR, which is hypothesized to be involved in host immune evasion (De Villiers and Zur, 2008). As shown previously, we observed variable amino acid residues across the entire ORF1 (Arze et al., 2021). However, we ascertained that most changes occurred within the HVR in contigs observed in multiple longitudinal samples from the same individual. This suggests a continuously evolving virus that might underly the high interpersonal diversity of the anellovirus community.

The observed members of the *Flaviviridae* belonged to the HPgV, a frequently identified member of the human blood virome. The incidence of HPgV infection has been estimated at 3–9% in the healthy population (Yang et al., 2020; Koonin et al., 2021). Initially, this virus was recognized as hepatitis G virus and associated with liver inflammation (Chivero and Stapleton, 2015). Additional data refuted these findings and could not further relate the presence of HPgV to any of the pathological conditions. Infection with HPgV has been associated to an altered immune response against pathogens. For instance, an immunomodulatory effect of HPgV has been observed in HIV infected individuals, which delayed the progression to AIDS (Tillmann et al., 2001; Lanteri et al., 2015; N’Guessan et al., 2017). Furthermore, coinfection of HPgV with Ebola virus decreased the mortality risk in infected patients (Lauck et al., 2015). Here, we observed the presence of HPgV in two patients diagnosed with liver cirrhosis and one patient with chronic hepatitis B. Remarkably, both liver cirrhosis patients reported regression of the disease during follow-up. Initially, liver cirrhosis was thought to be an irreversible remodeling of liver tissue. However, studies have shown that a successful suppression of the underlying cause of inflammation can lead to cirrhosis regression (Hytiroglou and Theise, 2018; Panel et al., 2021). The included study population is too limited to draw conclusive results. Nevertheless, our findings and previous reports indicate that this group of viruses deserves further attention in the HBV infection background.

The strength of this study was that we included longitudinal samples of chronic HBV infected cases, instead of cross-sectional sampling which is widely used in blood virome studies. This allowed a detailed assessment of virome dynamics in function of time. Furthermore, the access to a comprehensive clinical dataset enabled a thorough analysis of potential variables affecting the virome structure. The study was limited by the patient samples size and its retrospective nature of sample selection. Furthermore, interindividual differences could affect the comparability of virome dynamics in chronic HBV infected patients. Therefore, in future studies, we strongly advice to expand the patient sample sizes and consider homogenous cohorts or control specific variables that could affect the microbiome structure (e.g., geographical origin).

Viruses and their host interactions remain an enigmatic topic in the scientific world. In this study we have attempted to investigate the role of the blood virome in chronic HBV infected individuals. We have detected a variety of well-known viral families in the blood. The virome presented a higher intra-host stability, suggesting a more personalized blood virome composition. However, external factors including geographical origin could also contribute to the interpersonal variety in the viral community structure. No associations were found between specific virome members and the clinical course of chronic HBV infection. Additional studies are required to further elucidate the role of commensal viruses in HBV infection and human health. Finally, external variables that affect the blood virome structure should by explored in future studies.

## Data availability statement

The raw reads generated and analyzed for this study can be found in the NCBI’s Sequence Read Archive (SRA) database under accession no PRJNA828357.

## Ethics statement

The studies involving human participants were reviewed and approved by Ethics committee research UZ/KU Leuven. The patients/participants provided their written informed consent to participate in this study.

## Author contributions

MRP designed the study. MRP, DC, and FN recruited the patients and collected the samples and clinical data. MT, LVE, MNA, and MRP performed the sequencing and analysis of sequencing data. MT did the statistical analysis over the clinical and virome data. MT and FT provided the developed the first draft of manuscript. MT, FT, DC, MNA, LVE, FN, MVR, JM, and MRP edited the manuscript and improved it. All authors contributed to the article and approved the submitted version.

## Funding

MT (grant number 1S47118N) and LVE (grant number 1S25720N) are fellows at the Research Foundation Flanders (FWO, Belgium). MRP is supported by a postdoctoral grant from the FWO (1521716N). The funders had no role in the study design, data collection, data analysis, interpretation, and writing of the report. This study was supported by Gilead Sciences (grant number BE-2019-000016).

## Conflict of interest

The authors declare that the research was conducted in the absence of any commercial or financial relationships that could be construed as a potential conflict of interest.

## Publisher’s note

All claims expressed in this article are solely those of the authors and do not necessarily represent those of their affiliated organizations, or those of the publisher, the editors and the reviewers. Any product that may be evaluated in this article, or claim that may be made by its manufacturer, is not guaranteed or endorsed by the publisher.

## Abbreviations

dbRDA: distance-based redundancy analysis
HBeAb: hepatitis B e antibody
HBeAg: hepatitis B e antigen
HBsAb: hepatitis B s antibody
HBsAg: hepatitis B s antigen
HBV: hepatitis B virus
HCV: hepatitis C virus
HPgV-1: human pegivirus-1
ORF: open reading frame
OTU: operational taxonomical unit
PCA: principal component analysis
WTA: whole transcriptome amplification
