# Supplementary figures and images for: Plasma virome dynamics in chronic hepatitis B virus infected patients
Source: Front Microbiol. 2023 May 9;14:1172574. doi: 10.3389/fmicb.2023.1172574 (PMC10203228; doi:10.3389/fmicb.2023.1172574)

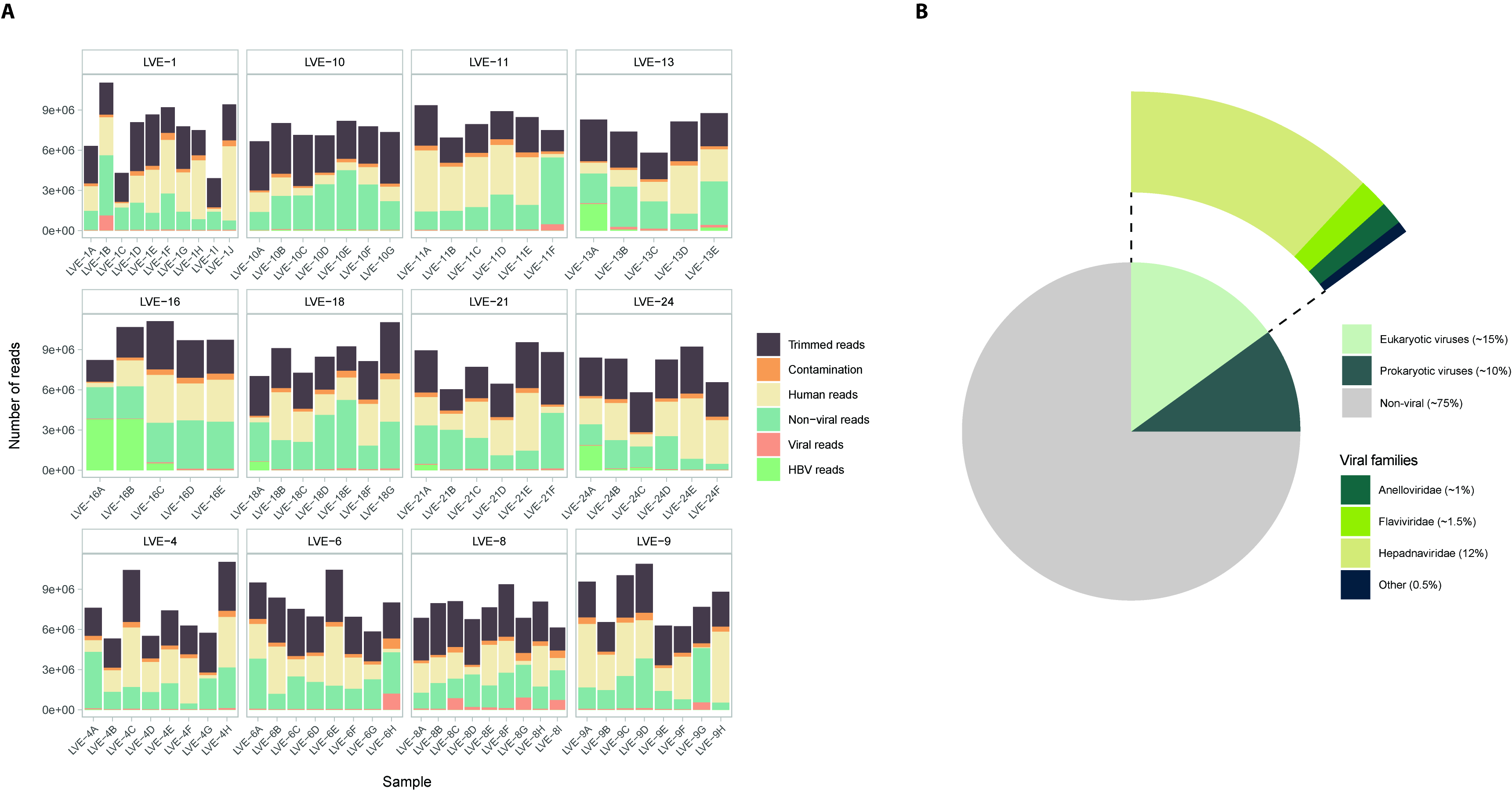

Supplement: Supplementary file 1 [file Image_1.TIF]

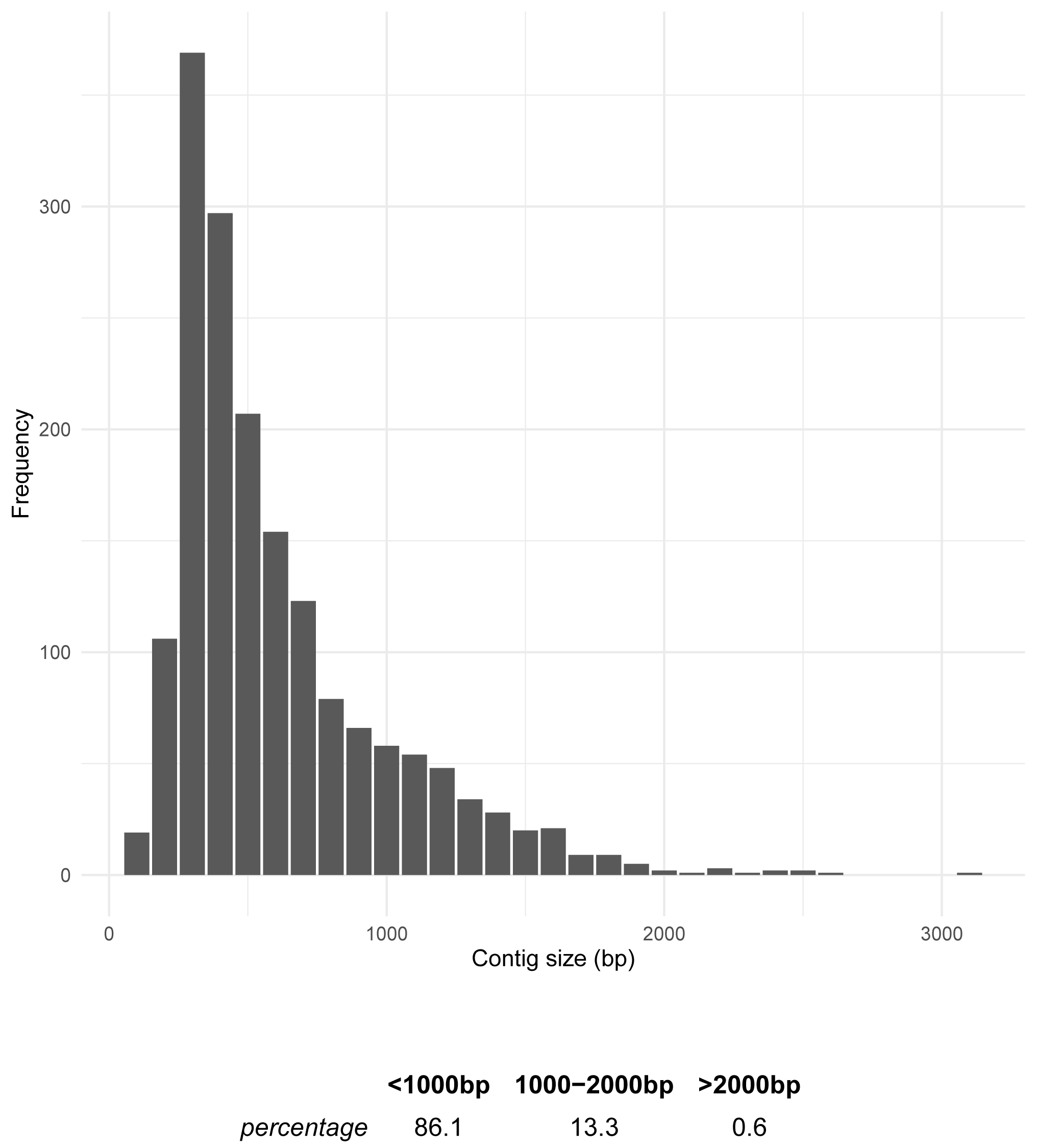

Supplement: Supplementary file 2 [file Image_2.TIF]

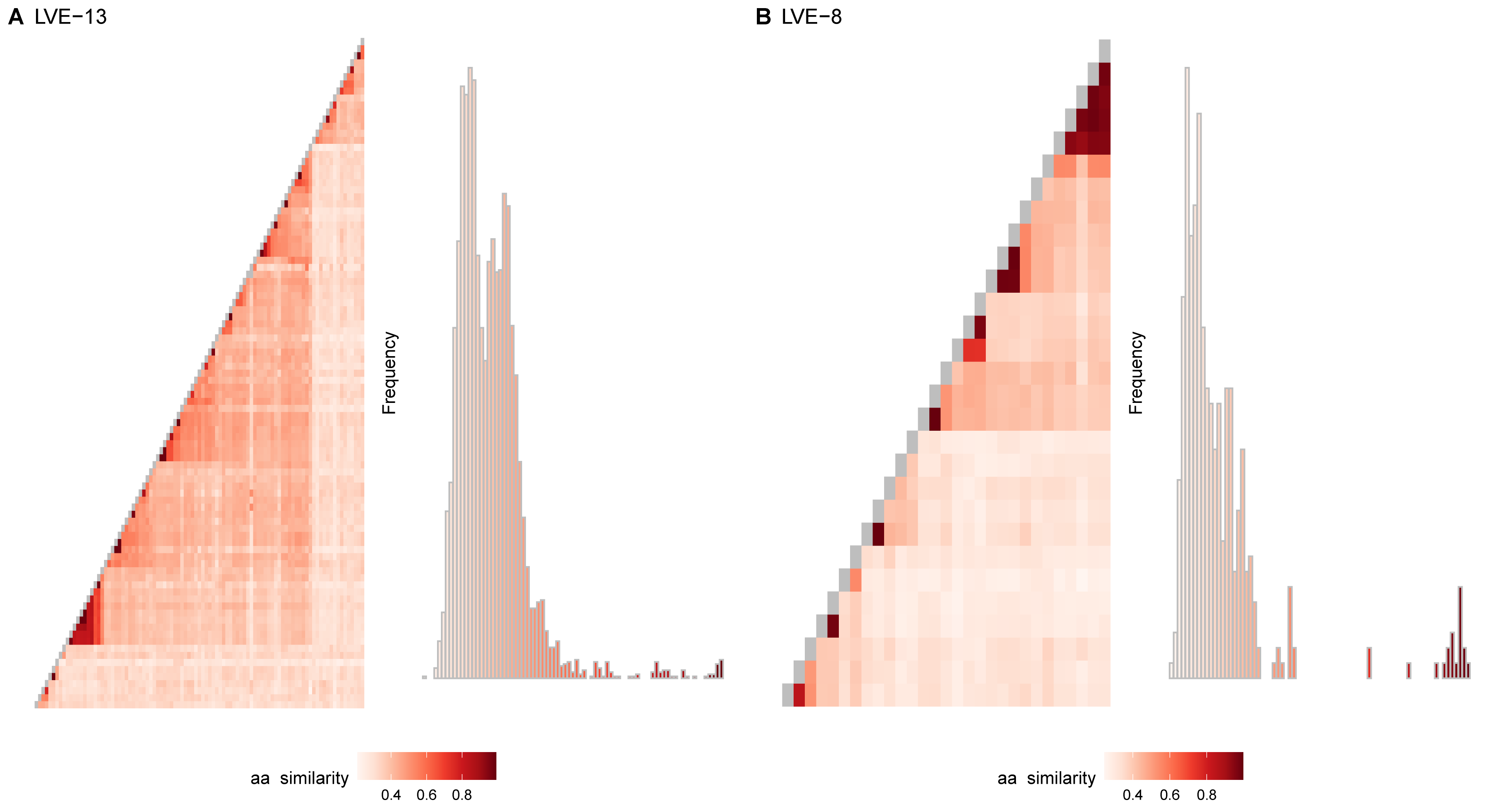

Supplement: Supplementary file 3 [file Image_3.TIF]

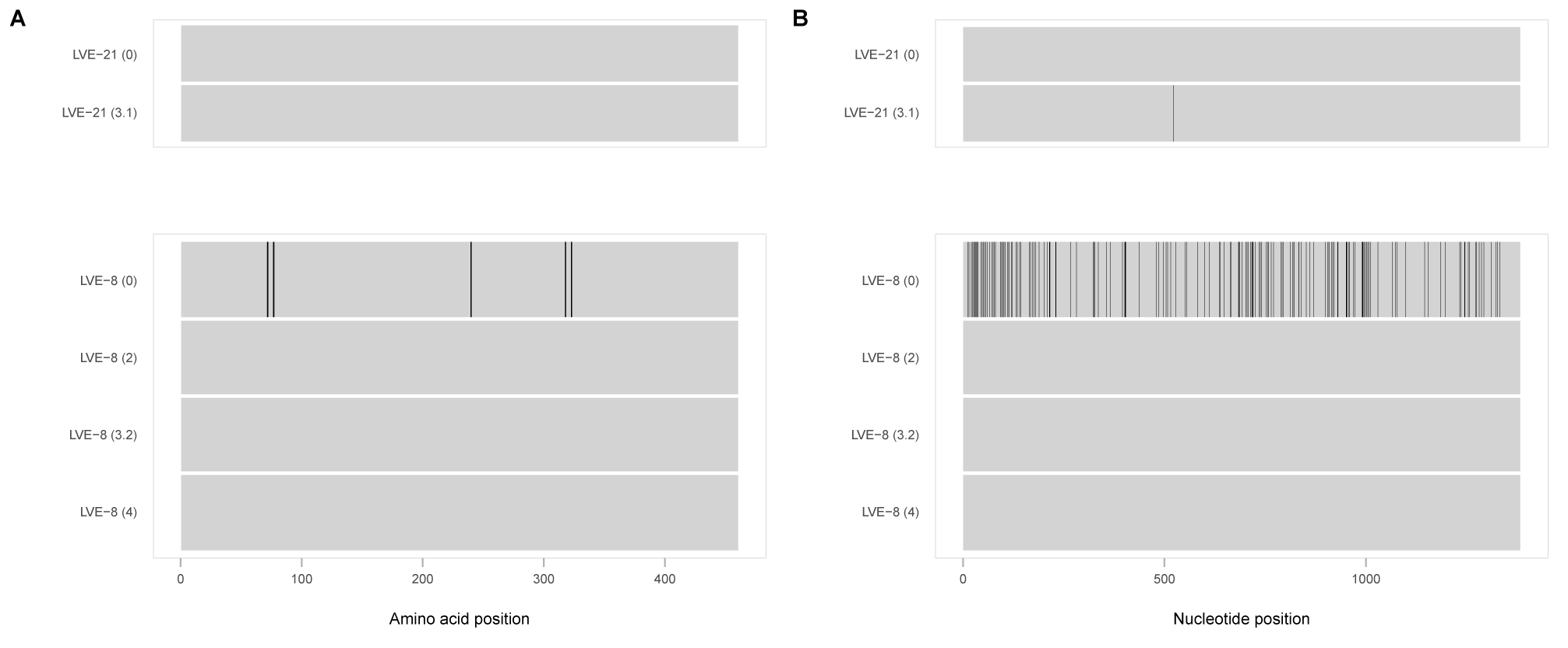

Supplement: Supplementary file 4 [file Image_4.TIF]
